# Explosive synchronization as a process of explosive percolation in dynamical phase space

**DOI:** 10.1038/srep05200

**Published:** 2014-06-06

**Authors:** Xiyun Zhang, Yong Zou, S. Boccaletti, Zonghua Liu

**Affiliations:** 1 Department of Physics, East China Normal University, Shanghai, 200062, China; 2 CNR- Institute of Complex Systems, Via Madonna del Piano 10, 50019 Sesto Fiorentino, Florence, Italy

## Abstract

Explosive synchronization and explosive percolation are currently two independent phenomena occurring in complex networks, where the former takes place in dynamical phase space while the latter in configuration space. It has been revealed that the mechanism of EP can be explained by the Achlioptas process, where the formation of a giant component is controlled by a suppressive rule. We here introduce an equivalent suppressive rule for ES. Before the critical point of ES, the suppressive rule induces the presence of multiple, small sized, synchronized clusters, while inducing the abrupt formation of a giant cluster of synchronized oscillators at the critical coupling strength. We also show how the explosive character of ES degrades into a second-order phase transition when the suppressive rule is broken. These results suggest that our suppressive rule can be considered as a dynamical counterpart of the Achlioptas process, indicating that ES and EP can be unified into a same framework.

Abrupt phase transitions have been observed in a variety of networked systems^1^, from epileptic seizures in the brain^2^ to cascading of power grids^3^ and jamming in the Internet^4^, and coping with them has been (and is currently) one of the most challenging problems. Recently, the occurrence of such transitions has been connected with the issue of percolation, i.e. the formation of a macroscopic connected component in the network. Explosive percolation (EP) was interpreted by a simple mechanism, equivalent, in fact, to a modified Erdős-Rényi (ER) growth process, where a product rule or sum rule (the so-called Achlioptas growth process) is additionally imposed^5^ which tends to suppress the formation of a large cluster before criticality. Since its putting forward, EP was immediately confirmed in regular lattice networks^6^ and scale-free (SF) networks^7^,^8^, and attracted a lot of interest in different contexts^9^,^10^,^11^,^12^,^13^,^14^,^15^,^16^,^17^,^18^,^19^. Whether EP is an exact first-order or a second-order transition in the thermodynamic limit^9^,^10^,^11^,^12^,^13^ is still matter of debate, and recently a stochastic model was designed to show that in the thermodynamic limit the EP transition can be either continuous or discontinuous^20^.

Together with the great progress in EP, it has been argued that also synchronization transitions on SF networks can occur explosively, i.e. by a discontinuous transition, called explosive synchronization (ES)^21^. Based on Kuramoto oscillators, ES has rapidly become a subject of enormous interest^22^,^23^,^24^,^25^,^26^,^27^,^28^,^29^,^30^,^31^,^32^. While originally it was suggested that ES was due to a positive correlation between the natural frequencies of oscillators and the degrees of nodes^21^, more recent studies have highlighted that ES can be also observed in homogeneous, non-SF, networks, provided that a positive correlation between the natural frequency of oscillator and its coupling strength exists^33^. Interestingly, these two kinds of positive correlations can be unified within the framework of mean-field, where the effective couplings are weighted to be proportional to the frequency of the oscillators^30^,^33^.

Notice that EP describes the abrupt change of the network structure, while ES denotes the abrupt change of the network's dynamical behavior. Uncovering the common features between EP and ES would then be extremely helpful to understand the various phenomena of abrupt transitions observed in natural phenomena. It is the aim of this work to propose a synthesization of EP and ES into a common framework.

## Results

### Description of the model

We start considering a network of *N* Kuramoto-like phase oscillators, whose evolution is ruled by: 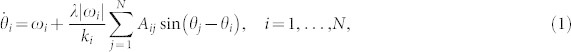
where dot denotes temporal derivative, *ω_i_* (*θ_i_*) is the natural frequency (the instantaneous phase) of oscillator *i*, *λ* is the overall coupling strength, 
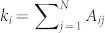
 is the degree of node *i*, and *A_ij_* are the elements of the symmetric adjacency matrix *A* (*A_ij_* = 1 when the nodes *i* and *j* are connected, and *A_ij_* = 0 otherwise). The model (1) can be considered as a frequency-weighted network and reflects the feature of several natural and social systems such as, for instance, power grid networks and communication networks. In the former case, a power grid network can be described as a network of Kuramoto oscillators, where the weighted coupling coefficient between two oscillators is related to their own natural frequencies^34^,^35^. In the latter case, an extrovert will contact his or her neighbors more frequently than an introvert. If we define the contact between two individuals as a kind of coupling and the frequency of contacts as coupling strength, the coupling strength will be then correlated with the characteristics of individuals, i.e. a kind of natural frequency of human being^35^.

The model (1) actually encompasses the degree-weighted case of Ref. 21 when the network is SF and *ω_i_* = *k_i_*. Ref. 33 demonstrated that ES can be observed in Eq. (1) for an arbitrary network topology, provided that the distribution *g*(*ω*) from which the natural frequencies of the network's oscillators are drawn is symmetric around zero. In what follows we will assume (unless when otherwise specified) *g*(*ω*) to be a Gaussian distribution with zero average and unit standard deviation.

### The global and local order parameters

**Figure 1:**
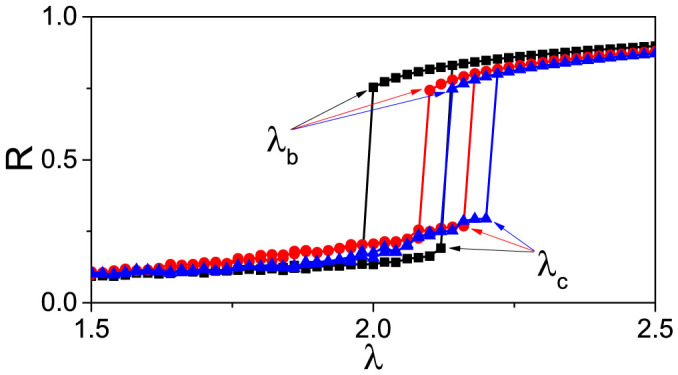
Synchronization transitions for different network's topologies. The three curves with “squares”, “circles” and “triangles” represent the cases of fully connected, ER and UCM networks, respectively, and *λ_c_* and *λ_b_* denote the critical points of forward and backward transition. The network size is *N* = 200 for all the three cases, and the average degree for both ER and UCM networks is 〈*k*〉 = 24.

The degree of phase coherence of the collective motion in Eq. (1) can be quantified by well known Kuramoto order parameter *R* defined by 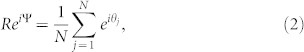
where 0 ≤ *R* ≤ 1 and Ψ denotes the average phase. Figure 1 shows the synchronization diagrams for *N* = 200, and for the cases of fully connected (squares), ER (circles) and SF (triangles) networks, the latter originating from the uncorrelated configuration model (UCM) with a power-law degree distribution^36^. From Fig. 1 one can see that there is an abrupt transition with an associated hysteretic loop in each of the three cases, which is produced by increasing *λ* adiabatically in both the forward and backward directions. The forward critical couplings for the three networks are *λ_c_* = 2.13, 2.17 and 2.21, respectively. Notice that the order parameter *R* remains at a small value before the jump, indicating that an increase in the coupling strength before *λ_c_* does not induce a giant cluster of phase synchronization.

**Figure 2:**
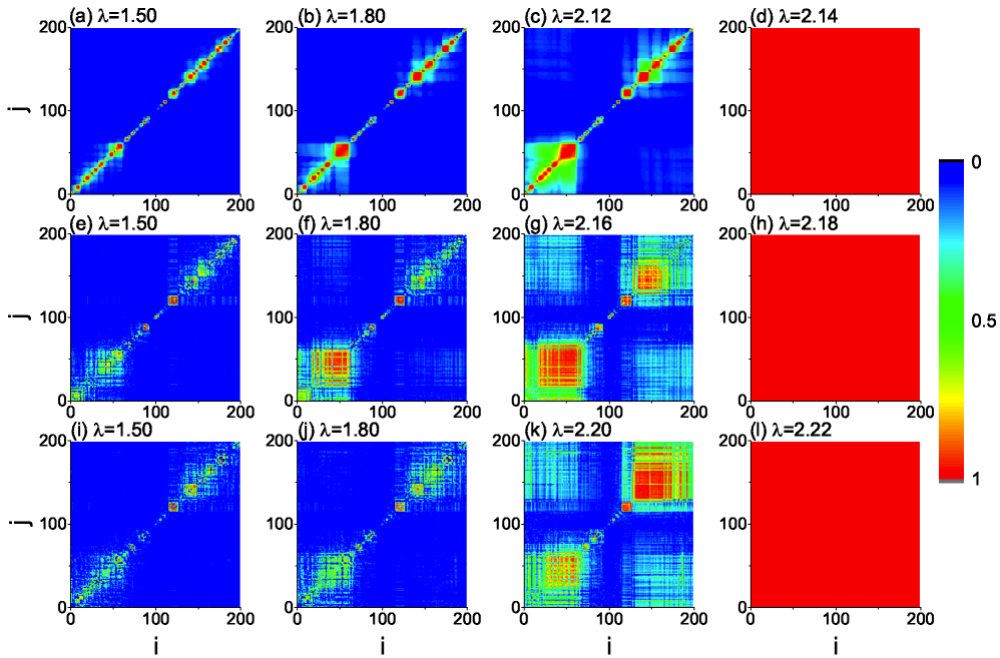
Plots of the matrix *R_ij_* for fully connected (first line), ER (second line) and UCM (third line) networks, where the oscillator *i* is labeled by the ascending order of frequency *ω_i_*. The coupling strengths are *λ* = 1.5, 1.8, 2.12, and 2.14 in (a) – (d) (*λ_c_* = 2.13); *λ* = 1.5, 1.8, 2.16, and 2.18 in (e) – (h) (*λ_c_* = 2.17); and *λ* = 1.5, 1.8, 2.20, and 2.22 in (i) – (l) (*λ_c_* = 2.21).

To gather more detailed information on the mechanisms underlying the observed scenario, we refer to the local order parameter, *R_ij_*, as defined in METHODS. *R_ij_* will be 1 for any two phase-locked oscillators, zero for all pairs of fully uncorrelated oscillators, and will take a value between 0 and 1 for any two partially correlated oscillators. Figures 2 (a)–(d) report the values of *R_ij_* for the fully connected network and for four typical *λ* values: namely (a) corresponds to *λ* = 1.5, (b) to *λ* = 1.8, (c) to *λ* = 2.12, i.e. right before *λ* = *λ_c_* = 2.13, and (d) to *λ* = 2.14, i.e. right after *λ_c_*, where the oscillator *i* is labeled by the ascending order of frequency *ω_i_*. It is interesting to notice that there are only small synchronized clusters of oscillators for *λ* < *λ_c_* (Fig. 2(a) to (c)), while a giant synchronized cluster shows up suddenly right after *λ_c_* (Fig. 2(d)), indicating that the small synchronized clusters are not gradually merged into larger synchronized clusters before *λ_c_*, but suddenly merge together right at *λ* = *λ_c_*. The very same scenario occurs for ER networks (Fig. 2(e) to (h)) and for UCM networks (Fig. 2 (i) to (l)), and can also be observed at around the backward critical point *λ_b_* (reported in Fig. 1 of the Supplementary Material). Such a scenario is in striking contrast with the classical scenario leading to a second order smooth transition to global phase synchronization (where an initial core of phase locked oscillators progressively attracts more and more elements in the network), and exhibits many similarities with that produced by the Achlioptas process in EP, where the formation of a giant percolation cluster is prevented before the transition^5^.

To show an evidence on the similarity between the evolution of ES and the Achlioptas process in EP, we here transform the dynamical synchronization process into an equivalent percolation process. For this purpose, we introduce the concept of *synchronized link* to indicate a pair of nodes *i* and *j* with *R_ij_* ≥ 0.95. In this way, no synchronized links will be present in the network at *λ* = 0, whereas more and more synchronized links will appear with the increase of *λ*. Take the fully connected network in Fig. 2(a) – (d) as an example. We find that the new synchronized links are always generated among those nodes with the adjacent numbers of *i* and *j*, i.e. close *ω_i_* and *ω_j_*, and then all the generated synchronized links will form some separated clusters when *λ* increases. Remarkably, it is found that these small clusters grow, but remain independent with the further increase of *λ* before *λ* < *λ_c_* (where suddenly all the clusters merge together to form a giant one), thus pointing to the very same process as in EP. To show the evolution of such a dynamical EP process, we have realized a movie in the Supplementary Material, where a point in the plane *i* − *j* appears all times a new synchronized link is formed.

### Suppressive rule of ES

To understand the mechanism of Achlioptas process in dynamical phase space, we focus on the phase-locking between two oscillators as observed in Fig. 2. Our theoretical analysis (whose details are extensively reported in METHODS) shows that a necessary condition of phase-locking between two oscillators is given by 
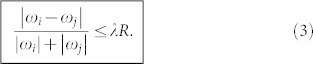


Let then 
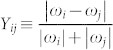
 be the frequency difference between nodes *i* and *j*, scaled by the factor |*ω_i_*| + |*ω_j_*|. *Y_ij_* will be 0 for *ω_i_* = *ω_j_*, 1 for opposite *ω_i_* and *w_j_*, while 0 < *Y_ij_* < 1 in any other case. Fig. 1 highlights that *R* takes a rather small value when *λ* < *λ_c_*, implying that only those pairs of oscillators with smaller *Y_ij_* can satisfy the condition (3) and thus form synchronized clusters. Since the scaling factor |*ω_i_*| + |*ω_j_*| represents the coupling weights of the two connected oscillators in Eq. (1), a smaller *Y_ij_* comes from a pair of oscillators with both larger couplings and smaller frequency difference.

On the other hand, a large value of *Y_ij_* will not satisfy the condition (3) and thus prevent synchronization between the corresponding oscillators. That is, the condition (3) will promote, at any *λ*, the synchronization between two connected oscillators with small *Y_ij_*, while suppressing the synchronization of those oscillators corresponding to large *Y_ij_*. This is the reason why the small synchronized clusters in Fig. 2 do not gradually merge together for *λ* < *λ_c_*: with the gradual increase of *λ*, those oscillators with smaller *Y_ij_* will firstly form multiple synchronized cores, and then the cores will attract their neighboring oscillators to form multiple synchronized clusters. Because of the uniformly distributed larger *Y_ij_*, the synchronized clusters cannot merge together into larger synchronized clusters, but only attract more and more neighboring free oscillators. When all the free oscillators have been attracted to the synchronized clusters, the further increase of *λ* cannot make the synchronized clusters become larger, but makes the clusters to merge each other suddenly, this way producing the significant jump on *R* observed in the explosive transition.

**Figure 3:**
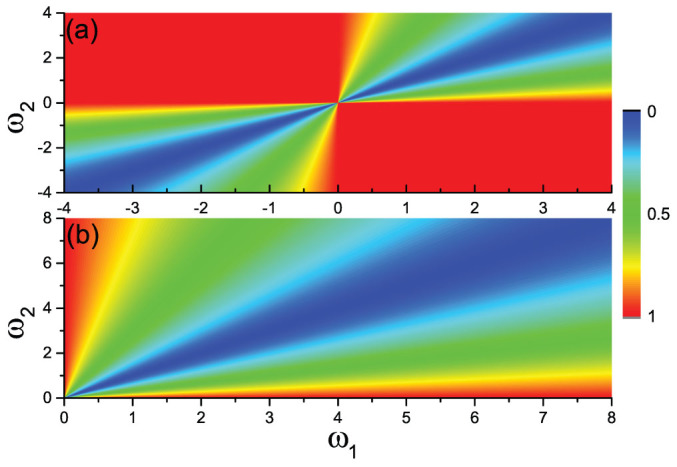
Plot of the matrix *Y_ij_* with (a) representing the case of a symmetric *g*(*ω*) with both positive and negative *ω* and (b) the case with only *ω* > 0.

We call Eq. (3) a *suppressive rule* for the formation of synchronized local clusters in ES, and the rest of this work is dedicated to discuss how Eq. (3) controls the mechanism behind the observed transition to synchronization. Figure 3(a), for instance, shows the values of *Y_ij_* for a symmetric distributed *g*(*ω*) with both positive and negative values of *ω*. It is easy to see that the second and fourth quadrants correspond to suppressive areas of synchronization, with *Y_ij_* ~ 1. Now, for the cases considered in Figs. 1 and 2, all frequencies *ω_i_* are randomly and uniformly distributed in the networks. Therefore, half of the network's connected pairs will display a positive and a negative *ω*, while the other half will be formed by nodes with the same sign (positive or negative) of *ω*. Those pairs of connected oscillators with the same sign in *ω* will, therefore, display relatively small value *Y_ij_* (and thus will tend to form phase synchronized clusters), while those pairs with opposite sign of *ω* will have *Y_ij_* ~ 1. These latter pairs, as in the Achlioptas process, will prevent small synchronized clusters to merge into a bigger one, i.e. they will suppress the formation of a giant cluster of synchronized oscillators before criticality.

### Degradation from a first-order to a second-order phase transition

An effective proof of validity consists in reporting how ES relaxes into a smooth, second-order, phase transition as soon as the suppressive rule (3) is broken. To that purpose, let us consider the ER network of Figs. 1 and 2 as an example, and artificially re-adjust the network by randomly exchanging the frequencies of two nodes *i* and *j*. As the two nodes have *k_i_* and *k_j_* neighbors respectively, such an exchange will ultimately influence all the values *Y_il_* (with 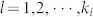 for the links between the node *i* and its neighbors) and *Y_jl_* (with 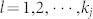 for the links between the node *j* and its neighbors).

Precisely, we introduce the quantity 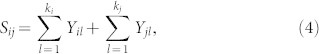
and we pick randomly a pair of nodes *i* and *j* in the network. If exchanging *ω_i_* and *ω_j_* would result in a value of *S_ij_* smaller than the original one, we accept the exchanging, while otherwise we make no operations. This way, one can gradually make more and more connected pairs with opposite signs in *ω* to turn into pairs displaying the same sign (negative or positive) of *ω*. Let us further introduce the quantity 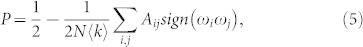
which represents the fraction of the pairs of connected oscillators with opposite sign in *ω*. *P* will take a value in between 0 and 1. Namely, *P* = 0.5 denotes the case of a uniform mixture, and *P* ≠ 0.5 implies that the uniform mixture is partially destroyed. With operating more and more of the proposed exchanging operations, one can gradually reduce *P*, until the case in which the network's oscillators are eventually divided into two big groups, with the same sign of *ω* for connected pairs belonging to the same group, and different signs of *ω* for connected pairs at the boundary of the two groups. Similarly, one can revert the acceptance condition for the exchange, and gradually increase *P* by artificially promoting links between oscillators with opposite signs of *ω*.

**Figure 4:**
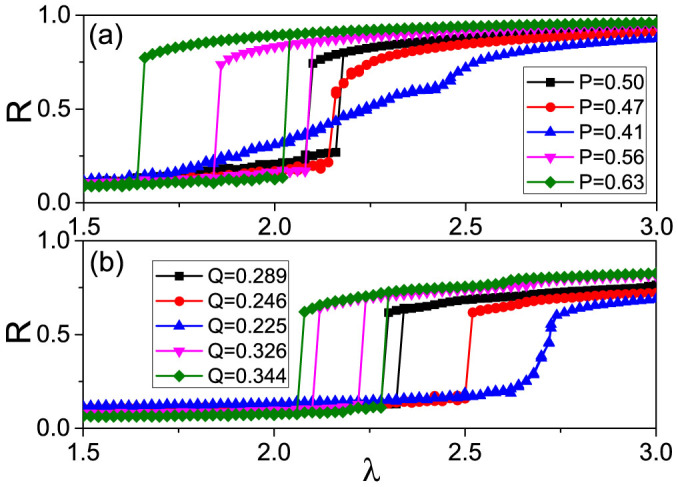
Synchronization diagrams obtained by operating frequency exchanges between connected nodes, as described in the text. (a) *g*(*ω*) taken as a Gaussian distribution with both positive and negative *ω*, where the “squares” denotes the case of no operation, “circles” and “up triangles” represent the cases of decreasing *P* to *P* = 0.47, and 0.41, respectively, and “down triangles” and “diamonds” represent the cases of increasing *P* to *P* = 0.56, and 0.63, respectively. (b) *g*(*ω*) taken as a Lorentzian distribution with only *ω* > 0. The “squares” denotes the case of no operation with *Q* = 0.289, “circles” and “up triangles” represent the cases of decreasing *Q* to *Q* = 0.246, and 0.225, respectively, and “down triangles” and “diamonds” represent the cases of increasing *Q* to *Q* = 0.326, and 0.344, respectively.

Figure 4(a) reports the transitions to synchronization observed at different levels of exchanging. Precisely, the curve with “squares” corresponds to the case of no operation, those with “circles” and “up triangles” represent the cases of decreasing *P* to *P* = 0.47, and 0.41, respectively, and those with “down triangles” and “diamonds” represent the cases of increasing *P* to *P* = 0.56, and 0.63, respectively. It is easy to see that decreasing *P* initially destroys the hysteretic loop (see the curve with “circles”), and eventually produces a transition with no jump (see the curve with “up triangles”), i.e. converting ES into a second-order phase transition. At variance, the sizes of the hysteretic loop for the “down triangles” and “diamonds” curves in Fig. 4(a) are both larger than that of “squares” with uniform mixture, indicating that increasing of *P* always enhances ES. See Fig. 2 in the Supplementary Materials for a detailed report on the different paths to synchronization induced by the exchanging process.

The suppressive rule (3) can also be applied for asymmetric frequency distributions, as, for instance the case of taking only the positive half of *g*(*ω*)^21^. Fig. 3(b) shows the matrix of *Y_ij_* with only *ω* > 0. From Fig. 3(b) we see that the value of *Y_ij_* is small nearby the diagonal, but close to unity in the red triangle area along the axes, indicating that the suppressive area of synchronization is formed here by those pairs of oscillators with one displaying a low frequency and the other displaying a large frequency. As the area of red triangle is proportional to the range of *ω*_2_(*ω*_1_), a larger range of *ω* (such as that arising from a SF distribution) will have the result of warranting the presence of a finite fraction of *Y_ij_* inside the suppressive area of synchronization. We here choose a Lorentzian distribution (
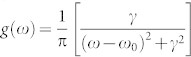
 with *ω*_0_ = 0, *γ* = 0.2 and *ω* > 0), which is an approximate power-law distribution. The existence of a sufficiently large suppressive area will prevent the merging of small synchronized clusters into a giant synchronized cluster before criticality, and thus we can observe ES (see the existence of the hysteretic loop in the “squares” curve of Fig. 4(b), obtained for the ER network and the Lorentzian distribution of *g*(*ω*) in Figs. 1 and 2).

Instead of *P*, we here introduce the quantity 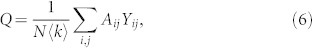
that measures the average connection between nodes with large and small *ω*, and repeat the exchanging process driven by Eq. (4), this way decreasing or increasing *Q*. Figure 4(b) shows the resulting synchronization transitions at different values of *Q*. Similarly to Fig. 4(a), the progressive decreasing of *Q* has the effect of eventually leading to a second-order transition, while increasing *Q* makes larger and larger the size of the hysteresis loop, thus enhancing ES.

## Discussion

The character of the phase transition accompanying explosive percolation has been the object of a continuous debate. At variance, there is no debate on the irreversible nature of ES because of the existence of an associated hysteretic loop. Yet, little was known about the robustness of ES so far. Here we have developed analytical tools to address this problem, and found that the robustness of ES depends on both the network topology and the frequency distribution. Moreover, we reveal that the suppressive rule (3) is the bridge between the first-order and second-order transitions and can be intuitively expressed as 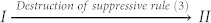

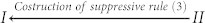
where *I* and *II* represent the first-order and second-order transitions, respectively.

Abrupt phase transitions are ubiquitous in both nature and man-made systems and can be understood from different perspectives. A natural but important question concerns the relationship between EP and ES, as the answer to such question will open a new window to understand the classical percolation transitions in complex networks. Our key finding, the suppressive rule (3), suggests that ES and EP can be unified in a same framework. Namely, for ES, a larger scaled frequency difference between two connected oscillators prevents their phase synchronization, and further suppresses, before the critical point, the merging of small synchronized clusters into a giant cluster. Similarly to the EP transition in configuration space, the process of forming synchronized clusters in ES can be considered as a dynamical Achlioptas process in phase space. According to the suppressive rule (3), the previous observations of ES from either a positive correlation between node's degree and frequency or a positive correlation between node's coupling strength are now well understood in terms of the existence of a suppressive area. Once the suppressive rule (3) is broken, ES returns to be a second-order phase transition. Our findings are therefore of relevance for controlling ES, by properly manipulating the distribution of frequencies on nodes.

On their turn, our results raise new questions. First of all, based on the unified framework of the suppressive rule (3), what is the counterpart system in the ES perspective, which corresponds to the continuous/discontinuous debate in the EP systems? Second, what happens when more complicated frequency distributions are considered such as the double peaks Lorentzian distribution of *g*(*ω*)? Finally, can our result apply to the real dynamics of networks in situations such as epileptic seizures? We predict that our work will stimulate further research efforts on these and related challenging issues.

## Methods

To measure the phase correlation between nodes *i* and *j*, we introduce the local order parameter 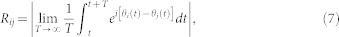
where *T* is the time window to measure the correlation. *R_ij_* will be in between 0 and 1 and a larger value of *R_ij_* represents a stronger phase synchronization. We use this approach to produce the Fig. 2 and the Figs. 1 and 2 in the Supplementary Material. Similarly, we can also use other approaches to measure the phase correlation between nodes *i* and *j*. For example, we may choose the cross correlation 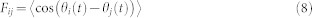
where 〈.〉 represents the average on time. The Fig. 3 in the Supplementary Material is produced by Eq. (8).

The suppressive rule (3) can be derived as follows. We first consider the case of a fully connected network. From Eq. (2), we have 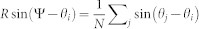. Plugging into Eq. (1), one obtains 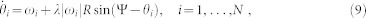
where *f*(*λ*, *ω_i_*) ≡ *λ*|*ω_i_*|*R* represents the effective coupling. Then, we consider the case of non-fully-connected networks. For an uncorrelated network, we follow Refs. 23, 37 to rewrite Eq. (1) as 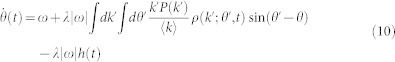
where *P*(*k*), 〈*k*〉, *ρ*(*k*; *θ*, *t*) represent the degree distribution, average degree, and density of the nodes with phase *θ* at time *t* for a given degree *k*, respectively, and the term *h*(*t*) takes into account time fluctuations and is given by 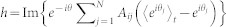, where “Im” stands for the imaginary part. We may regard *h* as a sum of *k* approximately uncorrelated terms and thus expect *h* to be of order 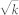. Under the assumptions of *k* ≫ 1 and substantially above the transition the term h(t) in Eq. (10) can be neglected. On the other hand, when this approximation is applied to numerical examples where the finite-size effect is not small, the theory is still useful in that it correctly indicates the trend of the numerical observations^37^. Correspondingly, Eq. (2) can be rewritten as 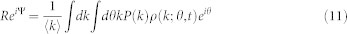
which gives 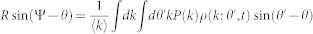. Neglecting the fluctuation *h*(*t*) and substituting Eq. (11) into Eq. (10), we obtain 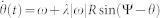, which is exactly Eq. (9). Therefore, we have Eq. (9) for the cases of both fully connected and non-fully-connected networks. Ref. 33 demonstrated that all the cases of ES can be unified within the mean-field framework of (9), provided that *f*(*λ*, *ω_i_*) is proportional to the natural frequency of the oscillators. We therefore take here Eq. (9) as our starting point. The evolution of the phase difference Δ*θ_ij_* ≡ *θ_i_* − *θ_j_* is then given by 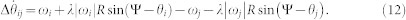
When the two oscillators *i* and *j* are phase-locked, one has 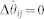, and 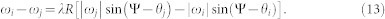
The maximum value of the right hand side of Eq. (13) is *λR*(|*ω_i_*| + |*ω_j_*|), which gives a necessary condition of phase-locking between two oscillators, i.e. the suppressive rule (3).

## Supplementary Material

Supplementary Information Explosive synchronization as a process of explosive percolation in dynamical phase space

Supplementary Information Explosive synchronization as a process of explosive percolation in dynamical phase space
