# Supplementary material for: Explosive synchronization as a process of explosive percolation in dynamical phase space
Source: Sci Rep. 2014 Jun 6;4:5200. doi: 10.1038/srep05200 (PMC4650870; doi:10.1038/srep05200)
Supplement: Supplementary Information — Explosive synchronization as a process of explosive percolation in dynamical phase space [file srep05200-s2.pdf]

Explosive synchronization as a process of explosive percolation in dynamical phase space

Xiyun Zhang[1], Yong Zou[1], S. Boccaletti[2] and Zonghua Liu[1]

1 Supplementary Figures

[ht]

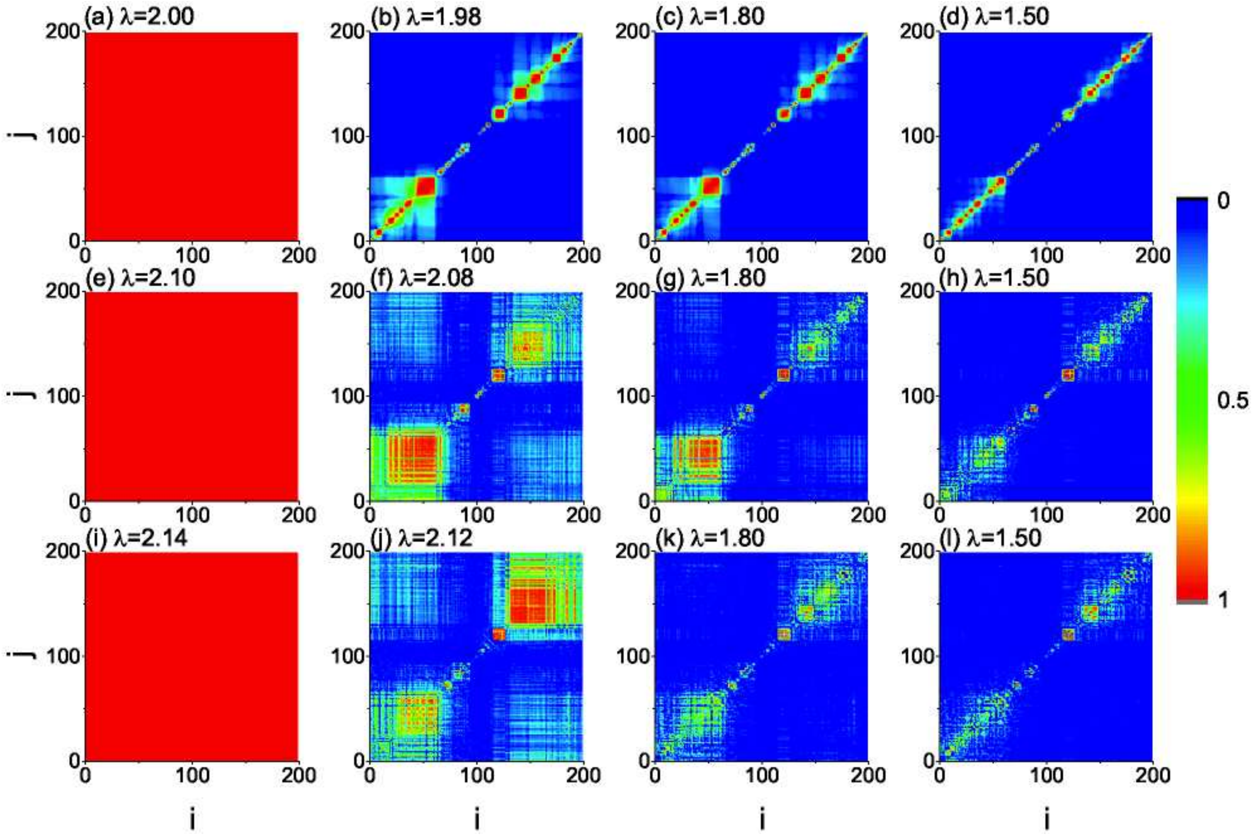

[ht]

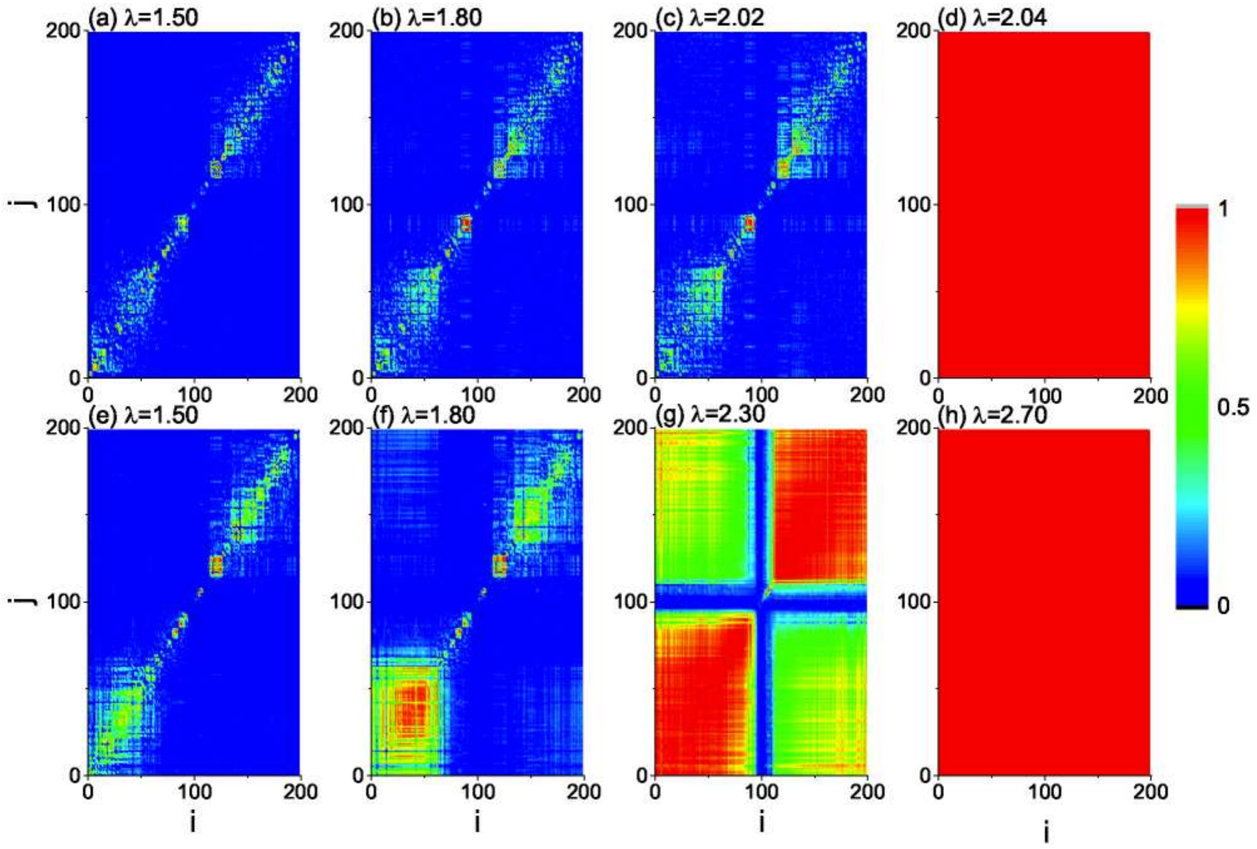

(a) λ=1.50
(b) λ=1.80
(c) λ=2.02
(d) λ=2.04
(e) λ=1.50
(f) λ=1.80
(g) λ=2.30
(h) λ=2.70
j
i
0
100
200
1
0.5
0

[ht]

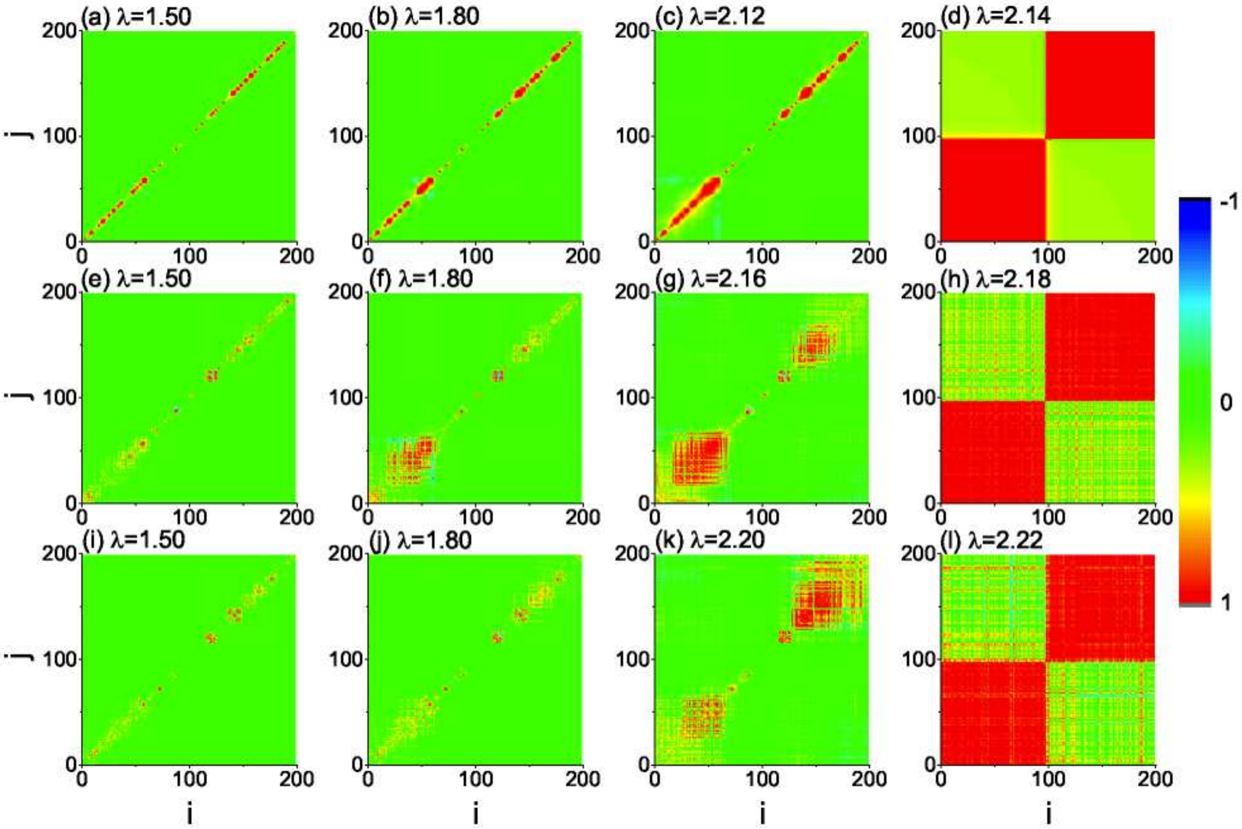

2 Supplementary Movie

Figure 1 (Color online.) **Case of backward transition corresponding Fig. 2 in main text**. Plots of the matrix R_{ij} for fully connected (first line), ER (second line) and UCM (third line) networks, where the oscillator i is labeled by the ascending order of frequency ω_i. The coupling strengths are $\lambda = 2.0, 1.98, 1.8$, and 1.5 in (a)-(d) ($\lambda_c = 1.99$); $\lambda = 2.1, 2.08, 1.8$, and 1.5 in (e)-(h) ($\lambda_c = 2.09$); and $\lambda = 2.14, 2.12, 1.8$, and 1.5 in (i)-(l) ($\lambda_c = 2.13$).

Figure 2 (Color online.) **Local order parameter R_{ij} for the case of varying P (see main text for definitions).** Data to be compared with Fig.4(a) of the main text. First line: Plots of the matrix R_{ij} with $P = 0.63$ and $\lambda_c = 2.03$, where the coupling strengths are set to be $\lambda = 1.5$ (a), $\lambda = 1.8$ (b), $\lambda = 2.02$ (c), and $\lambda = 2.04$ (d). Second line: Plots of the matrix R_{ij} with $P = 0.41$, where the coupling strengths are set to be $\lambda = 1.5$ (e), $\lambda = 1.8$ (f), $\lambda = 2.3$ (g), and $\lambda = 2.7$ (h). Notice that, in (e)-(h), the small original synchronized clusters gradually merge together to form a giant synchronized cluster, indicating a second-order transition.

Figure 3 (Color online.) **Measuring cross correlation by $F_{ij} = \langle \cos(\theta_i - \theta_j) \rangle$.** Plots of the matrix F_{ij}, for fully connected (first line), ER (second line) and UCM (third line) networks. The coupling strengths are $\lambda = 1.5, 1.8, 2.12$, and 2.14 in (a)-(d) ($\lambda_c = 2.13$); $\lambda = 1.5, 1.8, 2.16$, and 2.18 in (e)-(h) ($\lambda_c = 2.17$); and $\lambda = 1.5, 1.8, 2.20$, and 2.22 in (i)-(l) ($\lambda_c = 2.21$).
